# Nutritional Composition and Productivity of *Panicum maximum* cv. “Mombasa” Under Different Levels of Nitrogen Fertilization and Water Deficit

**DOI:** 10.3390/life14121614

**Published:** 2024-12-05

**Authors:** Saleh Alsunaydi, Abdulaziz B. Alharbi, Abdulrahman A. Al-Soqeer, Mohamed I. Motawei

**Affiliations:** 1 Department of Plant Production, College of Agriculture & Food, Qassim University, Buraydah 52571, Saudi Arabia; salehshot@gmail.com; 2 Department of Environment & Natural Resources, College of Agriculture & Food, Qassim University, Buraydah 52571, Saudi Arabia; 3 The National Research and Development, Center for Sustainable Agriculture, Estidamah, Saudi Arabia

**Keywords:** *Panicum maximum* cv. Mombasa grass, plant, water stress, fertilization, nutritional composition, productivity

## Abstract

This study investigates the production and nutritional quality of *Panicum maximum* cv. Mombasa grass under varying levels of water stress and nitrogen (N) fertilization, aiming to enhance forage production in harsh environments. Four irrigation levels (5760, 6912, 4608, and 3456 m^3^ ha^−1^ year^−1^) and three N fertilizer doses (115, 57.5, and 0 kg ha^−1^ year^−1^) were tested. The results indicate that Mombasa grass produced higher fresh and dry weights under higher irrigation levels (I1 and I2) compared to water deficit conditions across all cuts. Interestingly, under moderate water stress (I3), the dry weight was not significantly different from that under higher irrigation for the sixth harvest in the first season. Water deficit conditions led to a significant reduction in protein content across all treatments. However, under lower irrigation levels (I3 and I4), there was a significant increase in phosphorus (P), potassium (K^₊^), iron (Fe^2₊^), and zinc (Zn) concentrations. A heatmap analysis of shape descriptors grouped the productivity and nutritional traits into two clusters based on their response to combined fertilization and drought stress. This analysis revealed that the dry weight, number of leaves, and Fe and Zn contents were positively affected under moderate water stress (80% of control; 4608 m^3^ ha^−1^ year^−1^) with recommended N fertilization. The study concludes that *Panicum maximum* cv. Mombasa is tolerant to moderate water stress and is suitable for forage production in the Qassim region, Saudi Arabia.

## 1. Introduction

Panicum Guinea grass (*Panicum maximum* cv. Mombasa) is a feed crop and includes several types and the best is named Maximum. Because of its high yield and nutritional value, *Panicum maximum* is significant for pasture, green silage, and production. This species can adapt to a variety of habitats and is grown in several countries [1]. The grass species *Panicum maximum* (syn. *Megathyrsus maximus*) presents considerable potential for the production of dry matter (DM) in subtropical and tropical environments and is known to reach an annual production of dry matter of around 33 t ha^−1^ [2]. *Panicum* has a 16% protein content overall, which aids in drying the crop and increases production to 10 tons/hectare per year based on a dry substance [3]. This crop has been discovered to significantly boost milk output in cows and is excellent for fat production in goats, sheep, and calves [4]. The proper management of soil fertility and knowledge of the nutritional requirements of *Panicum maximum* are essential for the practice of pasture management, which is reflected in higher productivity and availability of food for the animals [5,6,7,8].

N is an important constituent of proteins, maximizing the dry matter yield of forage grasses as well as being the main nutrient for the maintenance of their productivity [9]. It is assimilated by the plant and associated with carbon chains, promoting an increase in cellular constituents and consequently increasing the vigor of regrowth and the total green dry matter production of plants under favorable climatic conditions [10].

According to Euclides [5], several studies have demonstrated significant increases in the production of *Panicum maximum* following the supply of N. Although most of these experiments present linear responses, their magnitudes are varied. Thus, there is a need to develop research on N sources and their applied doses, focusing on the frequency and efficiency of their supply to better understand the productive response of forages in dry and rainy seasons [11]. N dosages impacted the nutritional value of the total plant, leaf blade, and stem. Increased N dosages resulted in increased crude protein levels that varied by approximately 10%. The nutritional value of Mombasa grass was positively affected by cutting heights and nitrogen dosages. During the wet season, it is advised to manage the Mombasa grass with a forage residue of 0.40 m and apply up to 300 kg.ha^−1^ of nitrogen [12].

Panicum can withstand droughts without needing large amounts of water for irrigation, yet it will grow more quickly if water is available. It yields twice as much as alfalfa while requiring only half as much water. Any irrigation method, including surface irrigation, drip irrigation, submersion irrigation, or spray irrigation, can be used to cultivate it [13]. However, water stress has been demonstrated to reduce biomass production in *P. maxima* by up to 44% [14], owing to photosynthetic stomatal and metabolic restrictions [15]. Water stress frequently reduces soil nitrogen uptake by fodder roots [16]. This lowers the N and P content of plant tissues, leading to an increase in the C:N and C:P ratios and a decrease in biomass output. However, the stoichiometric adjustment in the *P. maxima* growth response to the combination of water stress and nitrogen fertilization has not been studied to our knowledge [17]. With the purpose of helping to identify promising forage crops for arid areas in Saudi Arabia, this study aimed to determine the tolerance of *P. maxima* cv. Mombasa to water stress levels under doses of nitrogen fertilization. Specifically, the study sought to evaluate how different levels of nitrogen fertilization interact with water stress to affect nutrient allocation and plant production.

## 2. Materials and Methods

### 2.1. Site Description and Establishment of Trials

The experiment was conducted at the Qassim University Agricultural Research Station (26°18′28″ N, 43°46′ E) from August 2020 to July 2022. The physical and chemical properties of the soil texture (at 0–25 cm in depth), pH, EC, organic matter, total N, available P, K, Se, and Zn were analyzed according to Page [18] (Table 1).

### 2.2. Plant Material and Experimental Design

Seeds of *P. maximus* cv. “Mombasa” were sown in August 2020 for the irrigation and fertilization trials. The treatments consisted of a combination of four levels of irrigation levels (5760 (I1), 6912 (I2), 4608 (I3), and 3456 (I4) m^3^ ha^−1^ year^−1^) and three levels of nitrogen supply (115 (F1), 57.5 (F2), and 0 (F3) kg ha^−1^ yard^−1^), with the split-plot arrangement in a randomized complete block design (RCBD) with four replications, totaling 48 experimental units of 6 m^2^ each. The irrigation treatments are the main plots, and the fertilizer treatments are the split plots. Urea (45% N) was applied as the nitrogen fertilizer. Supplemental irrigation was provided during seedling growth and pasture establishment. After this initial period, the plants were cut 30 cm above the ground. Then, the irrigation and fertilization treatments began and were maintained for two seasons (2020/2021 and 2021/2022). Overall, there was almost one cut every month. During the experimental period, we performed four evaluations at three and six cuttings for each season.

### 2.3. Measurements

1—Fresh and dry weights

The plant material, collected from an area of 1 m^2^, was weighed and placed in paper bags, dried in an air circulation oven at a temperature of 65 °C until it reached a constant weight, and subsequently weighed to determine dry biomass production.

2—Chlorophyll content

Chlorophyll pigment was measured using a Minolta chlorophyll Meter SPAD-501 (Spectrum Technologies, Inc., Aurora, IL, USA).

3—Plant height

Three plants were selected randomly to measure plant height.

4—Number of shoots and leaves

Three plants were selected randomly to measure the number of shoots and leaves per plant.

5—Nutritional Determination

For the determination of N, P, K, Zn, and Fe values in forage, the samples were rinsed with deionized water and dried in the air for 25 min and then at 70 °C for 48 h. All samples were sieved to <0.15 and <0.5 mm using a stainless steel mill. One-gram quantities of each plant sample were digested with a mixture containing concentrated HNO_3_, H_2_SO_4_, and H_2_O_2_ [19]. In the digestion solution, Fe and Zn were measured using Atomic Absorption Spectrometry (Shimadzu 7000), while K was measured using a GENWAY PFP7 flame photometer, and P was calorimetrically determined using a spectrophotometer (Varian Cary 50 Bio UV-Vis) according to Chapman and Pratt [20]. The nitrogen content was measured using the Kjeldahl method (Buchi B-324 Kjeldahl Distillation Unit) [20]. The nitrogen in protein was converted to ammonium sulfate by H_2_SO_4_ during digestion. This salt, in the process of steam distillation, liberated ammonia, which was collected in a boric acid solution. It was titrated against standard acid until a violet color was recorded [21]. Phosphorus determination was determined as described by Chapman and Pratt [20]. The phosphate ion was determined using an ammonium molybdate, which reacted with phosphor to produce phosphor–molybdate. Finally, this solution was reduced to a molybdenum blue and photometrically measured at 880 nm.

### 2.4. Statistical Analyses

An analysis of variance (ANOVA) was performed using JMP Ver. 11 [22] to compare the means of the irrigation and fertilization treatments for all variables. A linear mixed model was used in which the irrigation and fertilization factors were regarded as fixed factors. Significant differences among treatment means were calculated based on Duncan’s multiple range tests at *p* < 0.05. A heat map (HM) was generated using XLSTAT software version 2019 [23]. XLSTAT software was used to classify the data into different clusters to define Mombasa grass traits versus water stress levels and three doses of nitrogen fertilization.

## 3. Results

### 3.1. Effect of Irrigation and Fertilization Levels on Fresh and Dry Weights

Drought stress (I4) significantly reduced the fresh and dry weights of Mombasa for the four cuts (Table 2). At the high level of irrigation (I1 and I2), Mombasa grass gave higher fresh and dry weights compared to water deficit conditions for all cuts. Moreover, there were no significant differences between the control (I1) and the high level of irrigation (I2) for the dry weight of Mombasa for all cuts except harvest #3 in the second season. It should be noted that the dry weight of Mombasa under a water deficit (I3) was not significantly different from the high level of irrigation for harvest #6 in the first season.

Increasing the rates of nitrogen significantly increased the fresh and dry weights of Mombasa for all cuts (Table 2). Mombasa overall had significantly lower fresh and dry weights at 57.5 (F2) kg N ha^−1^ than 100 (F1) N kg ha^−1^.

**Figure 1:**
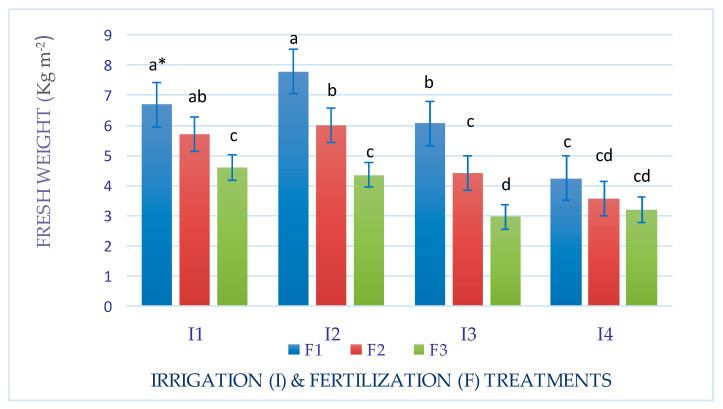
Fresh weight of Mombasa grass in harvest #3 under irrigation treatments and nitrogen fertilization doses in the 2020/2021 season. * Means separated by same lowercase letters (a, b, c, and d) in the figure were not significantly at *p* = 0.05.

**Figure 2:**
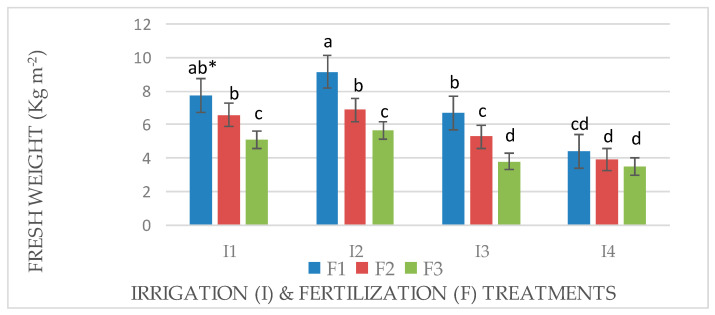
Fresh weight of Mombasa grass in harvest #6 under irrigation treatments and nitrogen fertilization doses in the 2020/2021 season. * Means separated by same lowercase letters (a, b, c, and d) in the figure were not significantly at *p* = 0.05.

**Figure 3:**
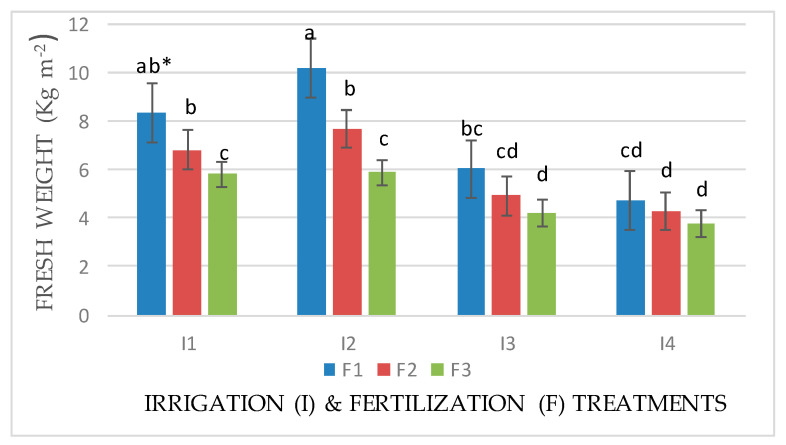
Fresh weight of Mombasa grass in harvest #3 under irrigation treatments and nitrogen fertilization doses in the 2021/2022 season. * Means separated by same lower-case letter (a, b, c, and d) in the figure were not significantly at *p* = 0.05.

**Figure 4:**
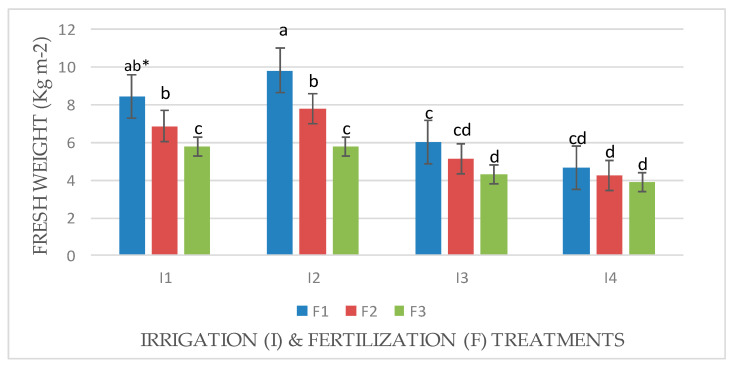
Fresh weight of Mombasa grass in harvest #6 under irrigation treatments and nitrogen fertilization doses in the 2021/2022 season. * Means separated by same lowercase letters (a, b, c, and d) in the figure were not significantly at *p* = 0.05.

**Figure 5:**
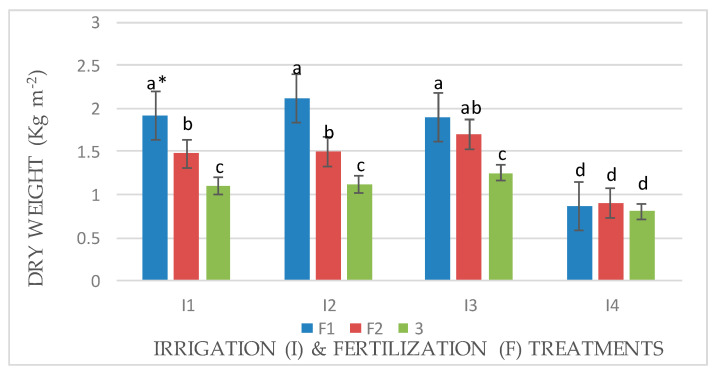
Dry weight of Mombasa grass in harvest #6 under irrigation treatments and nitrogen fertilization doses in the 2020/2021 season. * Means separated by same lowercase letters (a, b, c, and d) in the figure were not significantly at *p* = 0.05.

**Figure 6:**
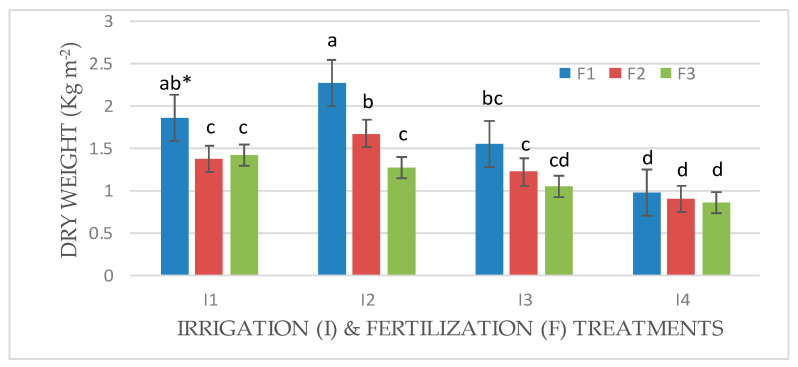
Dry weight of Mombasa grass in harvest #3 under irrigation treatments and nitrogen fertilization doses in the 2021/2022 season. * Means separated by same lowercase letters (a, b, c, and d) in the figure were not significantly at *p* = 0.05.

**Figure 7:**
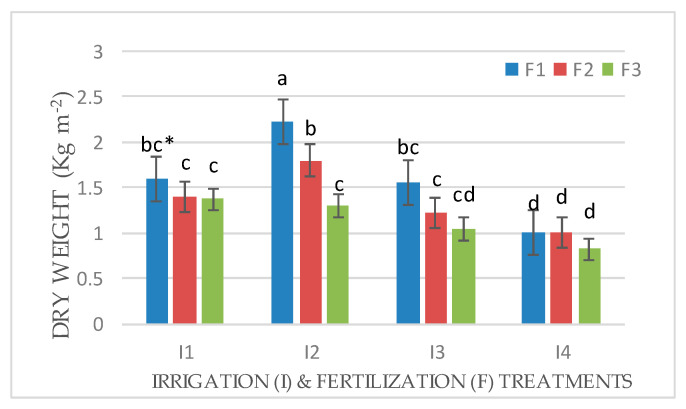
Dry weight of Mombasa grass in harvest #6 under irrigation treatments and nitrogen fertilization doses in the 2021/2022 season. * Means separated by same lowercase letters (a, b, c, and d) in the figure were not significantly at *p* = 0.05.

An interaction effect between factors (I × F) was observed for the fresh and dry weights of Mombasa in all cuts (Figure 1, Figure 2, Figure 3, Figure 4, Figure 5, Figure 6 and Figure 7). The best fresh and dry weights of Mombasa grass were obtained following irrigation at 6912 m^3^ ha^−1^ year^−1^ (I2) and fertilization at 115 kg N ha^−1^ yard^−1^ (F1) in all cuttings. However, in the sixth harvest under water stress (80% of control; 4608 m^3^ ha^−1^ year^−1^) and the high-level irrigation treatment with 115 kg N ha^−1^ yard^−1^ (F1), Mombasa grass showed a similar dry weight without significant differences in the first season (Figure 5). Moreover, Mombasa grass produced similar fresh and dry weights under both I1 and I2 with 115 kg N ha^−1^ yard^−1^ (F1) in harvest #3 in both seasons (Figure 1, Figure 2, Figure 3, Figure 4, Figure 5, Figure 6 and Figure 7).

### 3.2. *Effect of Irrigation and Fertilization Levels on Plant Height, Number of Leaves and Tillers, and Chlorophyll Content*

**Figure 8:**
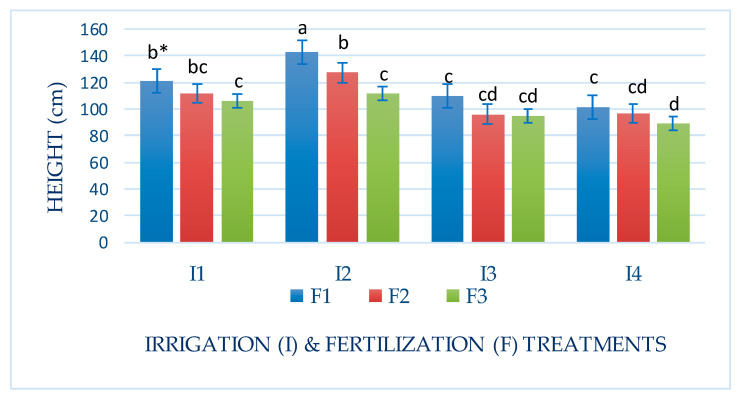
Plant height of Mombasa grass in harvest #3 under irrigation treatments and nitrogen fertilization doses in the 2020/2021 season. * Means separated by same lowercase letters (a, b, c, and d) in the figure were not significantly at *p* = 0.05.

**Figure 9:**
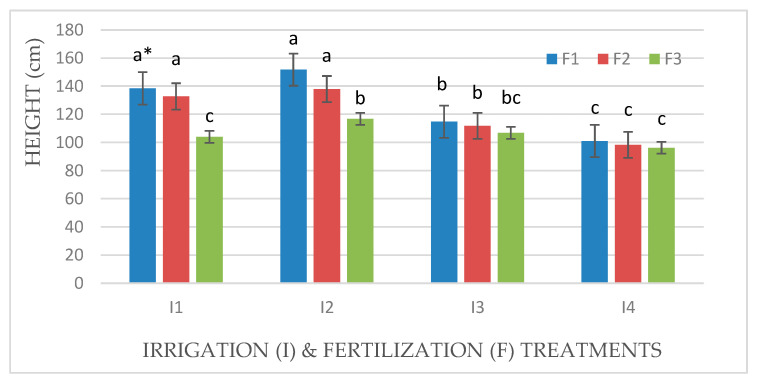
Plant height of Mombasa grass in harvest #6 under irrigation treatments and nitrogen fertilization doses in the 2020/2021 season. * Means separated by same lowercase letters (a, b, c) in the figure were not significantly at *p* = 0.05.

**Figure 10:**
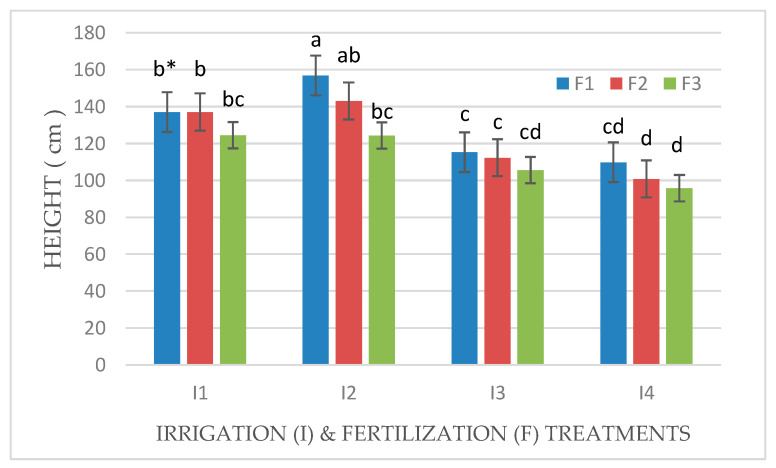
Plant height of Mombasa grass in harvest #3 under irrigation treatments and nitrogen fertilization doses in the 2021/2022 season. * Means separated by same lowercase letters (a, b, c, and d) in the figure were not significantly at *p* = 0.05.

**Figure 11:**
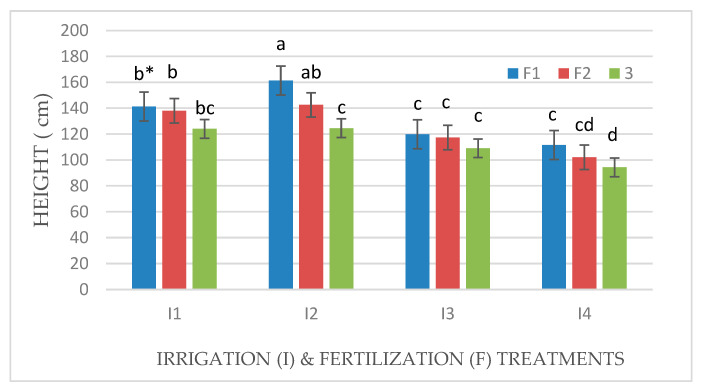
Plant height of Mombasa grass in harvest #6 under irrigation treatments and nitrogen fertilization doses in the 2021/2022 season. * Means separated by same lower-case letter (a, b, c, and d) in the figure were not significantly at *p* = 0.05.

Plant height and the number of leaves and tillers of Mombasa grass were significantly reduced under a water deficit (I4) for all cuts in both seasons (Table 2 and Table 3). Regarding the plant height of Mombasa grass, the highest level of irrigation (I2) yielded the highest Mombasa plant height in all cuts. Additionally, the Mombasa grass was the tallest under fertilization with 115 kg N ha^−1^ yard^−1^ (F1) in all cuts (Table 2 and Table 3). There was a significant effect of interaction between (I × F) for the plant height of the Mombasa grass (Figure 8, Figure 9, Figure 10 and Figure 11). The tallest Mombasa grass was observed under irrigation at 6912 m^3^ ha^−1^ year^−1^ (I2) and fertilization at 115 kg N ha^−1^ yard^−1^ (F1) in all cuttings.

Regarding the number of leaves and tillers of Mombasa grass, it was found that the highest number of leaves and tillers was obtained under high levels of irrigation (I1 and I2) in the first season. Additionally, the number of leaves and tillers of Mombasa grass under fertilization at 115 kg N ha^−1^ yard^−1^ (F1) was higher than those found in the other fertilization treatments in all cuts from both seasons. Regarding chlorophyll content, Mombasa grass under irrigation at 6912 m^3^ ha^−1^ year^−1^ (I2) showed the highest chlorophyll content in all cuts (Table 2 and Table 3). Under fertilization at 115 kg N ha^−1^ yard^−1^ (F1), the chlorophyll content of Mombasa grass was higher than that found in the other fertilization treatments in all cuts from both seasons.

### 3.3. Effect of Irrigation and Fertilization Levels on Nutritional Composition

The imposition of different water regimes caused a significant reduction in the protein content of Mombasa grass in all cuts (Table 4 and Table 5). This reduction was at a maximum following an irrigation treatment (I4) of 3456 m^3^ ha^−1^ year^−1^ for all cuts in both seasons. Under fertilization at 115 kg N ha^−1^ yard^−1^ (F1), the protein content of Mombasa grass was higher than that found in the other fertilization treatments in all cuts from both seasons (Table 4 and Table 5).

The water deficit conditions in irrigation treatments (I3 and I4) significantly increased shoot P, K^+^, Fe^++^, and Zn concentrations in Mombasa grass in all cuts except for the third harvest in the first season, when the Zn concentration was not significantly increased (Table 4 and Table 5). Nitrogen fertilization levels had no significant effect on shoot P, K^+^, Fe^++^, and Zn concentrations in Mombasa grass in the third harvest of both seasons except for P and K^+^ in the second season (Table 4 and Table 5). However, the control treatment yielded the highest concentrations of P, K^+^, Fe^++^, and Zn^++^ in Mombasa grass in the sixth harvest of both seasons.

**Figure 12:**
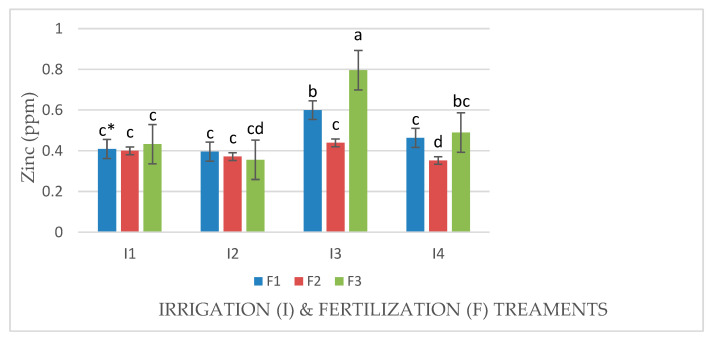
Concentrations of zinc (mg·kg^−1^) in Mombasa grass in harvest #6 under irrigation treatments and nitrogen fertilization doses in the 2020/2021 seasons. * Means separated by same lowecase letters (a, b, c, and d) in the figure were not significantly at *p* = 0.05.

**Figure 13:**
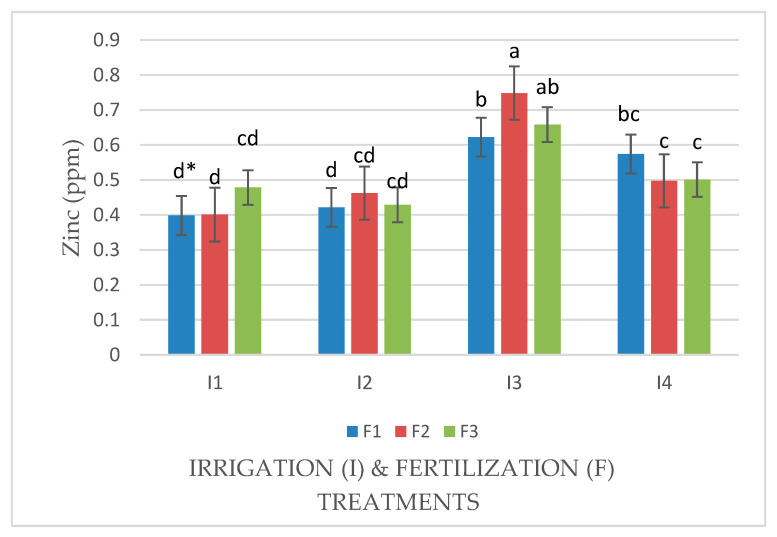
Concentrations of zinc (mg·kg^−1^) in Mombasa grass in harvest #6 under irrigation treatments and nitrogen fertilization doses in the 2021/2022 seasons. * Means separated by same lowecase letters (a, b, c, and d) in the figure were not significantly at *p* = 0.05.

An interaction effect between factors (I × F) was observed for the Zn concentration in Mombasa grass in the sixth harvest in both seasons (Figure 12 and Figure 13). In the absence of N, the Zn concentration of Mombasa grass in the sixth harvest was the highest under water stress (80% of control; 4608 m^3^ ha^−1^ year^−1^) in the first season. When N was supplied to the plant, the dose 57.5 kg ha^−1^ yard^−1^ (F2) gave the highest Zn concentration in Mombasa grass under water stress (80% of control; 4608 m^3^ ha^−1^ year^−1^) in the sixth harvest of the second season (Figure 13).

### 3.4. Heatmapping of Morph Quality Traits of Mombasa

**Figure 14:**
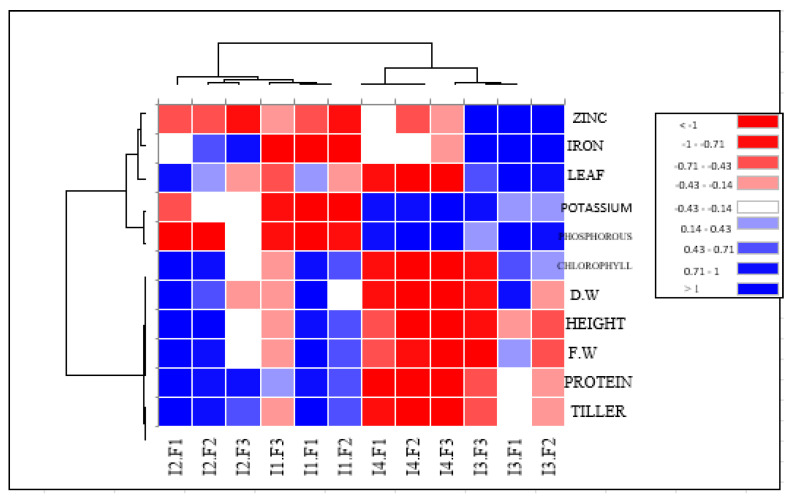
Heatmap cluster analysis dividing the productivity and nutritional composition of Mombasa traits into different clusters under water stress levels and nitrogen fertilization doses.

A heat mapping analysis indicated the relative performance of Mombasa grass under both the fertilization doses and water stress levels for which the data were recorded (Figure 14).

In the cluster heatmap analysis of shape descriptors, the productivity and nutritional composition of Mombasa traits were grouped into two major clusters according to responses to the combination of fertilization and drought stress (Figure 14). The first major cluster consisted of productivity traits, such as fresh and dry weights, the number of tillers, the protein content, and the chlorophyll content. The second cluster consisted of the nutritional composition of Mombasa grass traits. All traits illustrated different associations in Mombasa grass subject to water stress levels and nitrogen fertilization doses, varying from positive to negative extremes. It was observed that the dry weight, number of leaves, and zinc and iron contents of Mombasa grass had positive values under water stress (80% of control; 4608 m^3^ ha^−1^ year^−1^), and nitrogen fertilization was recommended. However, the other productivity traits of Mombasa grass had negative values under extreme water stress (60% of control).

## 4. Discussion

Water stress is known to directly reduce plant productivity [24]. In addition, water stress has a range of effects on the morphological, physiological, and biochemical processes of plants [25]. Our results show that water stress (I3 and I4) significantly reduced the fresh and dry weight of Mombasa for the four cuts. However, the dry weight of Mombasa under water deficit (I3) was not significantly different from the high level of irrigation for harvest #6 in the first season. Zuffo et al. [26] found that *P. maximum* cv. Mombasa grass has greater dry matter accumulation than the other forage grasses. In addition, Fonseca and Martuscello [27] reported that forage grasses belonging to the species *P. maximum* have a high potential for forage yield response to stressful environmental conditions.

Our results show that plant height and the number of leaves and tillers of Mombasa grass were significantly reduced under a water deficit (I4) for all cuts in both seasons. When a water deficit occurs, reductions in stomatal conductance and the photosynthesis rate lead to lower plant tillering potential [28]. Lower numbers of leaves and tillers in Mombasa grass under water stress conditions were also reported by Zuffo et al. (2022). In this study, the results regarding the effect of water stress on plant height are close to those of Purbajant et al. [29], who reported that the application of water stress has a significant effect on the plant height of *Panicum* grasses. In addition, the results show that Mombasa grass under irrigation at 6912 m^3^ ha^−1^ year^−1^ (I2) had the highest chlorophyll content in all cuts. However, the lowest chlorophyll content in Mombasa grass was found under water stress conditions (I4). This reduction in chlorophyll content under water stress conditions is a widespread phenomenon [30].

It was observed that water stress (I3 and I4) caused a significant reduction in the protein content of Mombasa grass in all cuts. According to [12], reductions in the crude protein content of the whole plant and the leaf blades of Mombasa were observed during the dry period. Generally, plants showing a small reduction in absorption nutrients are considered drought-resistant [31], but plant species and even genotypes within species may vary in their response to mineral uptake under water stress [32]. In the present study, water stress conditions during irrigation treatments (I3 and I4) significantly increased shoot P, K^+^, Fe^++^, and Zn concentrations in Mombasa grass in all cuts. Shahbaz et al. [33] concluded that increases in N, P, and K^+^ in blue panic grass were observed under water stress conditions.

This study demonstrated that increasing the rates of nitrogen significantly increased the fresh and dry weight of Mombasa for all cuts. The linear relation between nitrogen and dry mass production is indicative of the high potential of Mombasa grass to respond to this nutrient [8]. Cecato et al. [34] found that dry matter yields of Mombasa grass increased linearly with 200 kg N/ha/yr and were nearly twice those from pastures receiving no nitrogen. In addition, plant height and the number of leaves and tillers of Mombasa grass under fertilization at 115 kg N ha^−1^ yard^−1^ (F1) were higher than those found in other fertilization treatments in all cuts. The addition of nitrogen had interesting effects on the composition of forage, as increasing rates of N increased the numbers of both stems and leaves [3].

In our study, at a high nitrogen rate of 115 kg N ha^−1^ yard^−1^ (F1), the protein content of Mombasa grass was higher than that found when other fertilization treatments were used in all cuts. Linear increases in the crude protein production of Mombasa grass were observed by Munari-Escarela et al. [35] when studying the effect of N application rates. Hare et al. [3] suggested that applying 60 kg N ha^−1^ on each occasion appears to be a good compromise for achieving satisfactory dry matter yields, a leaf percentage higher than 70%, and a leaf crude protein concentration higher than 7%. Our results show that nitrogen fertilization levels had no significant effects on shoot P, K^+^, Fe^++^, and Zn concentrations in Mombasa grass in the third harvest. In addition, the control treatment of no N fertilization resulted in the highest concentrations of P, K^+^, Fe^++^, and Zn^++^ in Mombasa grass in the sixth harvest of both seasons. Decreased mineral content caused by increasing nitrogen doses is a controversial topic in the literature [11]. De Oliveira et al. [11] considered that increasing N doses were insufficient to promote changes in nutritional aspects. The cluster heat map analysis summarized the responses of the quality and productivity traits of Mombasa grass under both fertilization doses and water stress levels. All traits illustrated differential associations varying from positive to negative extremes in Mombasa grass under different water stress levels and nitrogen fertilization doses. It is interesting to note that the dry weight, number of leaves, and zinc and iron contents of the Mombasa grass had positive values under water stress (80% of control; 4608 m^3^ ha^−1^ year^−1^), and nitrogen fertilization was recommended. Zuffa et al. [26] suggested that *P. maximum* cv. Mombasa was identified as tolerant to water stress and has a high capacity for forage production under low soil water availability conditions.

In conclusion, our results suggest that while water stress poses significant challenges to Mombasa grass growth and productivity, nitrogen fertilization offers a promising strategy to mitigate the adverse effects of limited water supply and enhance forage quality and yield. Further research is needed to better understand the underlying mechanisms governing the response of Mombasa grass to water stress and nitrogen fertilization, which could inform more effective management practices in forage production systems in the future.

## 5. Conclusions

In conclusion, our study highlights the resilience of *Panicum maximum* cv. Mombasa under conditions of water stress and nitrogen fertilization, indicating its potential as a viable forage crop in arid regions. Water stress significantly affected the growth and physiological characteristics of Mombasa grass, reducing biomass production, chlorophyll content, and protein levels while increasing concentrations of certain minerals, such as phosphorus, potassium, iron, and zinc. This stoichiometric adjustment suggests that Mombasa grass adapts to water-limited environments by modulating nutrient uptake, which could contribute to its tolerance to drought conditions. Moreover, nitrogen fertilization had a positive impact on biomass, plant height, and protein content, with higher nitrogen levels generally enhancing these growth parameters. Interestingly, while nitrogen application improved yield and protein content, it did not consistently increase mineral concentrations. The combined effects of moderate water stress and optimal nitrogen fertilization rates resulted in improved quality and productivity traits, underscoring the adaptability of Mombasa grass to nutrient and water stress interactions. To strengthen these results, further research is needed across multiple growing seasons. Future studies should also explore additional variables, such as the effects of varying soil types, nutrient interactions, and long-term water stress adaptations. Expanding these perspectives will provide a more comprehensive understanding of the adaptability and resilience of Mombasa grass in water-limited environments.

## Figures and Tables

**Table 1:** Soil chemical and physical characteristics of the two seasons.

Season	Chemical Analysis						Physical Analysis (%)		
	K (ppm)	P (ppm)	N (ppm)	OM (%)	pH	EC (ds/m)	Clay	Silt	Sand
Season (2020–2021)	34	33.1	15.7	0.4	8.1	5.3	0.4	4.2	94.9
Season (2021–2022)	34.5	32.1	15.5	0.4	7.9	5.1	0.4	4.5	94.5

**Table 2:** The effect of irrigation treatments and nitrogen fertilization doses on fresh and dry weight, the number of tillers per plant, the number of leaves per plant, and the chlorophyll content in the 2020/2021 season.

TREATMENT		Season (2020–2021)											
		Harvest No. 3						Harvest No 6					
		F.W kg m^−2^	D.W kg m^−2^	HEIGHT cm	TILLER No.	LEAF No.	CHLOROPHYLL	F.W kg m^−2^	D.W kg m^−2^	HEIGHT cm	TILLER No.	LEAF No.	CHLOROPHYLL
**IRRIGATION**	**I1**	5.66 a	1.30 a	113 b	5.66 a	19.91 a	11.40 ab	6.48 b	1.5 a	125.08 b	14.41 a	52.33 a	10.96 b
	**I2**	6.07 a	1.47 a	127 a	5.66 a	25.25 a	12.48 a	7.23 a	1.58 a	135.50 a	14.41 a	59.08 a	12.38 a
	**I3**	4.48 b	1.05 b	100.08 c	5.16 a	25.0 a	10.08 b	5.25 c	1.54 a	111.08 c	13.16 b	62.66 a	10.23 b
	**I4**	3.66 c	1.05 b	95.66 c	4.00 b	12.91 b	7.80 c	3.95 d	0.85 b	98.50 d	9.91 c	31.83 b	7.70 c
**FERTILIZATION**	**F1**	6.22 a	1.50 a	118.68 a	5.62 a	24.75 a	11.71 a	7.00 a	1.70 a	126.50 a	14.06 a	59.43 a	11.60 a
	**F2**	4.91 b	1.15 b	107.93 b	5.18 a	21.81 a	10.60 b	5.66 b	1.33 b	120.18 b	12.62 b	49.18 b	10.48 b
	**F3**	3.78 c	1.00 b	100.18 c	4.56 b	15.75 b	9.00 c	4.53 c	1.06 c	105.93 c	12.25 b	45.81 b	8.88 c
I*F		**	**ns**	**	**ns**	**ns**	**ns**	**	**	**	**ns**	**ns**	**ns**

Means followed by the same letter in a column are not significantly different from each other at *p* = 0.05. ** Significant at *p* = 0.01. ns: Non-Significant at *p* = 0.05.

**Table 3:** The effect of irrigation treatments and nitrogen fertilization doses on the fresh and dry weights, the number of tillers per plant, the number of leaves per plant, and the chlorophyll content in the 2021/2022 season.

TREATMENT		Season (2021–2022)											
		Harvest No. 3						Harvest No 6					
		F.W kg m^−2^	D.W kg m^−2^	HEIGHT cm	TILLER No.	LEAF No.	CHLOROPHYLL	F.W kg m^−2^	D.W kg m^−2^	HEIGHT cm	TILLER No.	LEAF No.	CHLOROPHYLL
**IRRIGATION**	**I1**	6.99 b	1.55 b	132.83 b	27.58 b	91 bc	10.47 a	7.04 b	1.79 a	134.41 b	36.91 b	129.83 b	11.85 ab
	**I2**	7.90 a	1.74 a	141.33 a	31.33 a	111 b	11.08 a	7.86 a	1.77 a	142.75 a	40.75 a	147.75 b	10.85 a
	**I3**	5.05 c	1.27 c	110.33 c	23.16 c	141.8 a	9.89 b	5.15 c	1.27 b	115.33 c	33.08 c	183.75 a	9.95 bc
	**I4**	4.25 d	0.90 d	102.08 d	20.50 c	70.33 c	7.80 c	4.27 d	0.94 c	102.58 d	24.50 d	88.42 c	8.41 c
**FERTILIZATION**	**F1**	7.30 a	1.66 a	129.86 a	28.25 a	120.1 a	10.85 a	7.24 a	1.63 a	133.43 a	37.25 a	159.25 a	11.28 a
	**F2**	5.93 b	1.26 b	123.25 b	25.81 a	101.6 b	9.64 a	6.01 b	1.35 b	124.93 b	33.31 b	132.25 b	10.60 a
	**F3**	4.91 c	1.15 c	112.00 c	22.87 b	89 b	8.94 a	4.99 c	1.13 c	112.93 c	30.87 c	120.81 b	8.91 b
I*F		**	**	**	**ns**	**ns**	**ns**	**	**	**	**ns**	**ns**	**ns**

Means followed by the same letter in a column are not significantly different from each other at *p* = 0.05. ** Significant at *p* = 0.01. ns: Non-Significant at *p* = 0.05.

**Table 4:** The effect of irrigation treatments and nitrogen fertilization doses on protein %, phosphorus, potassium, iron, and zinc concentration in the 2020/2021 season.

TREATMENT		Season (2020–2021)									
		Harvest No. 3					Harvest No. 6				
		Protein (%)	P (ppm)	K (ppm)	Fe (ppm)	Zn (ppm)	Protein (%)	P (ppm)	K (ppm)	Fe (ppm)	Zn (ppm)
**IRRIGATION**	**I1**	16 b	783.9 b	53 b	780 c	0.4105 a	16 b	770.3 c	56.75 d	793.9 c	0.4140 b
	**I2**	18.2 a	887.9 b	73.2 a	3742 ab	0.3833 a	18.5 a	831 c	70.66 c	3824 ab	0.3749 b
	**I3**	12.5 c	2186.8 a	81.4 a	4570 a	0.5479 a	12.8 c	1521 b	79.50 b	4362.4 a	0.6117 a
	**I4**	10.5 d	1729 ab	77.1 a	2057 bc	0.3911 a	9.6 d	2072 a	96.4 a	2838 b	0.4353 b
**FERTILIZATION**	**F1**	15 a	1593 a	68.4 a	2537.7 a	0.4964 a	15.06 a	1140.7 b	72.37 b	2892.6 a	0.4672 b
	**F2**	14.3 a	1429 a	69.4 a	3022.1 a	0.4117 a	14.18 b	1205 b	71.87 b	3099.8 a	0.3910 b
	**F3**	13.6 b	1168 a	75.7 a	2837.7 a	0.3915 a	13.56 c	1549 a	83.25 a	2872.3 a	0.5185 a
I*F		ns	ns	ns	ns	ns	ns	ns	ns	ns	**

Means followed by the same letter in a column are not significantly different from each other at *p* = 0.05. ** Significant at *p* = 0.01, respectively. ns: Non-Significant at *p* = 0.05.

**Table 5:** The effect of irrigation treatments and nitrogen fertilization doses on protein %, phosphorus, potassium, iron, and zinc concentration in the 2021/2022 season.

TREATMENT		Season (2021–2022)									
		Harvest No. 3					Harvest No. 6				
		Protein (%)	P (ppm)	K (ppm)	Fe (ppm)	Zn (ppm)	Protein (%)	P (ppm)	K (ppm)	Fe (ppm)	Zn (ppm)
**IRRIGATION**	**I1**	16.08 b	616.6 b	52.5 c	2347.4 c	0.3963 b	16.91 b	803.25 d	59 d	2792.8 c	0.4259 c
	**I2**	18.08 a	653.6 b	71.3 b	3814.8 b	0.3875 b	18.00 a	1117.50 c	72 c	4715.2 b	0.4372 c
	**I3**	14.16 c	1785.3 a	83.9 a	4667.8 a	0.5571 a	15.83 c	1704.83 b	79.7 b	5706.8 a	0.6762 a
	**I4**	11.0 d	2218 a	90.6 a	3174.3 b	0.3830 b	12.16 d	2065.33 a	99.4 a	5214.8 b a	0.5242 b
**FERTILIZATION**	**F1**	16 a	1057.8 b	74.1 b	3723.4 a	0.4146 a	16.42 a	1274.5 b	73 b	4217.3 b	0.5040 a
	**F2**	14.50 b	1402.4 ab	69.5 b	3226.4 a	0.4442 a	15.37 b	1419.3 ab	79.4 a	4553.4 ab	0.5271 a
	**F3**	14 b	1495.2 a	80.1 a	3553.4 a	0.4341 a	15.37 b	1574.3 a	80.1 a	5051.5 a	0.5165 a
I*F		ns	ns	ns	ns	ns	ns	ns	ns	ns	**

Means followed by the same letter in a column are not significantly different from each other at *p* = 0.05. ** Significant at *p* = 0.01. ns: Non-Significant at *p* = 0.05.

## Data Availability

All datasets generated for this study are included in the manuscript.
